# A meta-collection of nitrogen stable isotope data measured in Arctic marine organisms from the Canadian Beaufort Sea, 1983–2013

**DOI:** 10.1186/s13104-021-05743-0

**Published:** 2021-09-06

**Authors:** Ashley Ehrman, Carie Hoover, Carolina Giraldo, Shannon A. MacPhee, Jasmine Brewster, Christine Michel, James D. Reist, Michael Power, Heidi Swanson, Andrea Niemi, Wojciech Walkusz, Lisa Loseto

**Affiliations:** 1 Fisheries and Oceans Canada, Central and Arctic Region, 501 University Crescent, Winnipeg, MB R3T 2N6 Canada; 2 Centre for Earth Observation Science, University of Manitoba, 125 Dysart Rd., Winnipeg, MB R3T 2N2 Canada; 3 Centre Manche—Mer du Nord, Ifremer, HMMN, BP 669, F-62 321 Boulogne sur Mer, France; 4 Biology Department, University of Waterloo, 200 University Ave. W, Waterloo, ON N2L 3G1 Canada

**Keywords:** Nitrogen stable isotopes, Trophic level, Marine ecosystem, Food web, Arctic

## Abstract

**Objectives:**

Existing information on Arctic marine food web structure is fragmented. Integrating data across research programs is an important strategy for building a baseline understanding of food web structure and function in many Arctic regions. Naturally-occurring stable isotope ratios of nitrogen (δ^15^N) and carbon (δ^13^C) measured directly in the tissues of organisms are a commonly-employed method for estimating food web structure. The objective of the current dataset was to synthesize disparate δ^15^N, and secondarily δ^13^C, data in the Canadian Beaufort continental shelf region relevant to trophic and ecological studies at the local and pan-Arctic scales.

**Data description:**

The dataset presented here contains nitrogen and carbon stable isotope ratios (δ^15^N, δ^13^C) measured in marine organisms from the Canadian Beaufort continental shelf region between 1983 and 2013, gathered from 27 published and unpublished sources with associated sampling metadata. A total of 1077 entries were collected, summarizing 8859 individual organisms/samples representing 333 taxa across the Arctic food web, from top marine mammal predators to primary producers.

## Objective

Severe and rapid climate-driven changes in temperature, sea-ice cover, nutrient delivery, and physical mixing dynamics have led to significant changes in species distributions, food webs, and ecosystem function across the Arctic [1, 2]. Existing information on Arctic marine food web structure is fragmented, such that integrating data across research programs is an important strategy for building a baseline understanding of food web structure and function in many Arctic regions.

Trophic level is a primary tenet of food web ecology that describes the vertical structure of food sources supporting top predators. Trophic level can be estimated from stable isotope ratios of nitrogen (δ^15^N) measured directly in consumer tissues because the heavy ^15^N isotope typically exhibits stepwise enrichment between predator and prey [3, 4]. The dataset presented here contains nitrogen stable isotope ratios (δ^15^N) measured in marine organisms from the Canadian Beaufort continental shelf region between 1983 and 2013, gathered from published and unpublished sources. Taxa are included from across all trophic levels, from top marine mammal predators to primary producers. The dataset was compiled for the purpose of cross-validating trophic level estimates calculated from a mass-balance food web model [5] with those calculated from observed δ^15^N values, to evaluate how well the model may capture ecosystem structure [6, 7]. However, δ^13^C data were also retained in the dataset where available because δ^15^N and δ^13^C are commonly used together in ecosystem and food web studies. The dataset may be of broad use for trophic and ecological investigations in Arctic marine ecosystems, either locally or incorporated into pan-Arctic studies.

## Data description

The dataset generated during the current study is available in the Polar Data Catalogue repository (CCIN Ref #13207), along with a detailed description of data collection methods and references for source publications (Table 1): https://doi.org/10.21963/13207 [8]. Readers should use version 2 of the available files (see Table 1).

**Table 1:** Overview of data files

Label	Name of data file/data set	File types (file extension)	Data repository and identifier
Data file 1	CBS_d15N_d13C_1983-2013_ReadMe_20210805_V2	Portable document format (.pdf)	Polar Data Catalogue, CCIN Ref #13207 (https://doi.org/10.21963/13207) [8]
Data file 2	CBS_d15N_d13C_1983-2013_Data_20210805_V2	Comma separated values (.csv)	Polar Data Catalogue, CCIN Ref #13207 (https://doi.org/10.21963/13207) [8]
Data set 1	CBS_d15N_1983-2013_References_20210105	Portable document format (.pdf)	Polar Data Catalogue, CCIN Ref #13207 (https://doi.org/10.21963/13207) (8)

A standardized search of primary literature was conducted to compile δ^15^N values measured in marine taxa captured on or near the Canadian Beaufort Sea continental shelf, including marine mammals, fish, benthic invertebrates, zooplankton, and primary producers, as well as organic matter and detritus. The Web of Science electronic database and Google Scholar were searched in 2018 using the phrases: ‘Beaufort Sea OR Western Arctic AND δ^15^N OR stable nitrogen’. Publications were discarded if they did not include raw or averaged δ^15^N data, did not report data for organisms captured on the Beaufort Sea Shelf and/or adjacent regions, did not cover the period from 2013 and earlier, or contained data already reported in another publication. To supplement primary literature sources, expert knowledge identified grey literature, dissertations, and unpublished sources containing δ^15^N data generated by field programs led by Fisheries and Oceans Canada, and in collaboration with the University of Waterloo (Waterloo, Ontario, Canada) and University of Manitoba (Winnipeg, Manitoba, Canada). If matching δ^13^C data were available from the resultant publications, they were added to the dataset. Note, however, that primary literature search terms did not include those specific to carbon stable isotopes, and therefore publications containing only δ^13^C data for the region were not included.

A total of 27 sources were retained, from which data were summarized as mean δ^15^N and δ^13^C by taxon, year, and/or sample site. The resulting dataset comprised 1077 entries, summarizing 8859 individual organisms/samples across 333 taxa, sediment, and detritus. All elemental stable isotope ratios (^15^N:^14^N, ^13^C:^12^C) were expressed in standard δ notation as parts per thousand (‰) relative to the international standard atmospheric N_2_ for nitrogen and Pee Dee Belemnite (or Vienne Pee Dee Belemnite) for carbon. Detailed methodologies for stable isotope analyses are outlined in the respective publications cited for each entry in the database. Raw data were summarized as presented numerically in the source publications; we did not perform any standardizations (e.g., isotopic baseline standardizations, mathematic lipid adjustments, etc.,) and did not estimate data from plots.

The following metadata were summarized for each entry whenever available: sample size, error (standard deviation, standard error, and 95% confidence limits), trophic level estimated by the source paper, tissue type analyzed, sampling location, sampling depth(s), sampling station(s), sampling year, and month/season. Information on sample pre-treatment that may influence interpretation of δ^15^N and δ^13^C data (e.g., acidification to remove inorganic carbon, lipid removal [13, 14]) was additionally captured in the dataset, albeit in simplified terms. Users should consult source publications when pre-treatment is relevant to their investigations. Taxonomic names provided in the original source publications were checked for updates or changes on the World Register of Marine Species [9]. The currently accepted scientific names, authority, and unique Aphia ID were recorded for each entry to increase taxonomic certainty for future applications.

Each entry was assigned to one of the trophic groups used in the existing Ecopath with Ecosim model (31 of 36 groups represented; [6, 10] A subset of the data pertaining specifically to the continental shelf (< 200 m depths) was used to calculate mean trophic level for each model group, weighted by sample size, using two commonly-employed stable isotope modelling approaches [11, 12]. Cross-validation indicated the Ecopath with Ecosim model generally performed well when compared to δ^15^N-derived trophic level estimates, but performance was poorer for trophic groups whose diets have not yet been well-characterized [7].

## Limitations

Data are summarized as mean values with associated error and metadata whenever available. In some cases, error, sample size, and/or some classes of metadata were not reported in the source publication and are thus missing from the dataset. See associated ‘Read Me’ file for details.

## Data Availability

The data described in this Data note can be freely and openly accessed on the Polar Data Catalogue under CCIN Reference #13207 at https://doi.org/10.21963/13207 [8]. Please see Table 1 and references for details and links to the data.
